# A Fast Algorithm for 2D DOA Estimation Using an Omnidirectional Sensor Array

**DOI:** 10.3390/s17030515

**Published:** 2017-03-04

**Authors:** Weike Nie, Kaijie Xu, Dazheng Feng, Chase Qishi Wu, Aiqin Hou, Xiaoyan Yin

**Affiliations:** 1 School of Information Science and Technology, Northwest University, Xi’an 710127, China; weikenie@163.com (W.N.); chase.wu@njit.edu (C.Q.W.); yinxy@nwu.edu.cn (X.Y.); 2 School of Electro-Mechanical Engineering, Xidian University, Xi’an 710071, China; kjxu@stu.xidian.edu.cn; 3 National Laboratory of Radar Signal Processing, Xidian University, Xi’an 710071, China; dzfeng@xidian.edu.cn; 4 Department of Computer Science, New Jersey Institute of Technology, Newark, NJ 07102, USA

**Keywords:** sensor array, fast algorithm, spectrum peak diffusion effect, Direction of Arrival estimation, 2D MUSIC

## Abstract

The traditional 2D MUSIC algorithm fixes the azimuth or the elevation, and searches for the other without considering the directions of sources. A spectrum peak diffusion effect phenomenon is observed and may be utilized to detect the approximate directions of sources. Accordingly, a fast 2D MUSIC algorithm, which performs azimuth and elevation simultaneous searches (henceforth referred to as AESS) based on only three rounds of search is proposed. Firstly, AESS searches along a circle to detect the approximate source directions. Then, a subsequent search is launched along several straight lines based on these approximate directions. Finally, the 2D Direction of Arrival (DOA) of each source is derived by searching on several small concentric circles. Unlike the 2D MUSIC algorithm, AESS does not fix any azimuth and elevation parameters. Instead, the adjacent point of each search possesses different azimuth and elevation, i.e., azimuth and elevation are simultaneously searched to ensure that the search path is minimized, and hence the total spectral search over the angular field of view is avoided. Simulation results demonstrate the performance characters of the proposed AESS over some existing algorithms.

## 1. Introduction

Direction of Arrival (DOA) is a critical parameter in many sensor-based systems using radar, sonar and wireless communications. As a well-known high-resolution DOA estimation algorithm, the MUltiple SIgnal Classification (MUSIC) algorithm [1] has attracted considerable attention in the areas concerned with estimating parameters from observations of array output [2]. However, this algorithm suffers from prohibitively high computational cost for its search-based strategy, especially for two dimensional (2D) DOA estimation.

The work by Huang [3] indicated that it is generally difficult to control a fixed stepsize in the adaptation process due to the inherent conflict between fast convergence and estimated accuracy. A large stepsize may simplify calculation at the expense of a worse Root Mean Square Error (RMSE); meanwhile, a small stepsize may require more calculation but with an improved RMSE. Variable stepsize algorithms [4] often include a coarse-grained search and a fine-grained search with a large and small stepsize respectively, which can effectively balance the convergence rate and error. By combining MUSIC results of two decomposed linear arrays, Zhou [5] accurately estimated the DOA with phase ambiguity removed. In its search procedures of two arrays, an adaptive scheme including coarse-grained and fine-grained search is used to reduce the complexity.

1D processing approaches can also be applied for solving the 2D DOA problem to reduce computational complexity. Wang [6] successfully used 1D MUSIC with a novel tree structure to estimate the azimuth and elevation dependently. By constructing a novel relation between azimuth and elevation, Karthikeyan [7] proposed an elegant 1D search technique to compute the 2D DOA estimation using the MUSIC algorithm in the case of a single source. A robust 2D DOA estimation based on two 1D angles estimations using L-shaped array is proposed by Kikuchi [8], and its pair matching of azimuth and elevation is accurate in low SNR and multi-source scenarios.

Search-free [9] methods are an important approach for reducing complexity. Han [10] reconstructed a special antenna array based on the Toeplitz matrix whose rank is only related to the DOA of the signals, hence the subspace can be extracted without being affected by the coherence between the signals. With the estimated subspace, an improved ESPRIT method is provided to implement the DOA estimation without spectrum peak search for coherent signal DOA estimation. Chen [11] extended the algorithm in [10] to a 2D situation and presented another search-free algorithm, where they obtained a block Hankel matrix from correlations and found that the rank of the block Hankel matrix only depends on the number of impinging waves. Two matrix pencils including DOA parameters are derived from the signal subspace of the Hankel matrix, and a pairing free method [12] is used to achieve the 2D parameters estimation. As a well-known search-free fast algorithm, Root-MUSIC [13,14] algorithm converted spectrum search into finding roots of a complex polynomial formed from noise subspace. Generally, Root-MUSIC is more accurate than the normal MUSIC in a larger stepsize such as 1°, but for a smaller stepsize such as 0.1°, the MUSIC algorithm yields a higher accuracy than Root-MUSIC. In the presence of completely polarized electromagnetic wave, Costa [15] proposed a joint high-resolution 2D DOA and polarization estimator [16,17] based on polynomial rooting techniques and Fast Fourier decomposition of the steering vector. Several antenna arrays including small handheld terminals and channel sounding applications show a significant improvement in performances.

Goossens and Rogier [18] proposed an efficient 2D DOA estimation algorithm based on the combination of UCA-RARE and Root-MUSIC. They estimated the azimuth angles by UCA-RARE which decoupled the estimation of azimuths from that of elevations. For each azimuth, they designed a new search-free rooting algorithm by expanding the array manifold into a double Fourier series. It is proved that even in the presence of severe mutual coupling [19,20], this hybrid approach can yield very robust 2D DOA estimation performances.

Converting complex-valued computations to real-valued ones can often accelerate DOA estimations. Ren [21] preprocessed the block Hankel matrix in [11] through an averaging like method for unitary transformation, which transforms the eigenvalue and singular value decomposition into real-valued computation. Subsequently, the DOA estimations are solved by 1-D unitary ESPRIT [22]. The 2D unitary ESPRIT algorithm in [23] involves a specific unitary transformation to formulate the eigenvalue decomposition in terms of a real-valued computation.

Motivated by these low-complexity algorithms, which have been successfully in applying sensor array processing [24], this paper proposed a fast 2D MUSIC algorithm, which involves three rounds of searching: the first two rounds are 1D searches along a circle and straight lines, respectively, and the third one is a 2D search on several concentric circles. The contribution of this work lies in the observation of spectrum peak diffusion effect and its utilization for finding the approximate direction of sources, while most of the other works in the literature concentrate on how to suppress this effect. The second contribution is the design of a search strategy to minimize the search path and avoid the total spectral search over the angular field of view.

Simulation results demonstrate that with the same search stepsize, AESS has a similar estimated RMSE compared with the 2D MUSIC algorithm, while the computing complexity and the actual running time are remarkably reduced. Meanwhile, with the same computation cost, the estimated RMSE of AESS is significantly improved. The 2D DOA estimation success rate is also analyzed versus the computation complexity. To further evaluate the estimation performance, we present the scatter plots of estimated 2D angle, and compare the running time of the proposed AESS with the 2D MUSIC algorithm and PIE algorithm.

## 2. Problem Formulation

**Figure 1:**
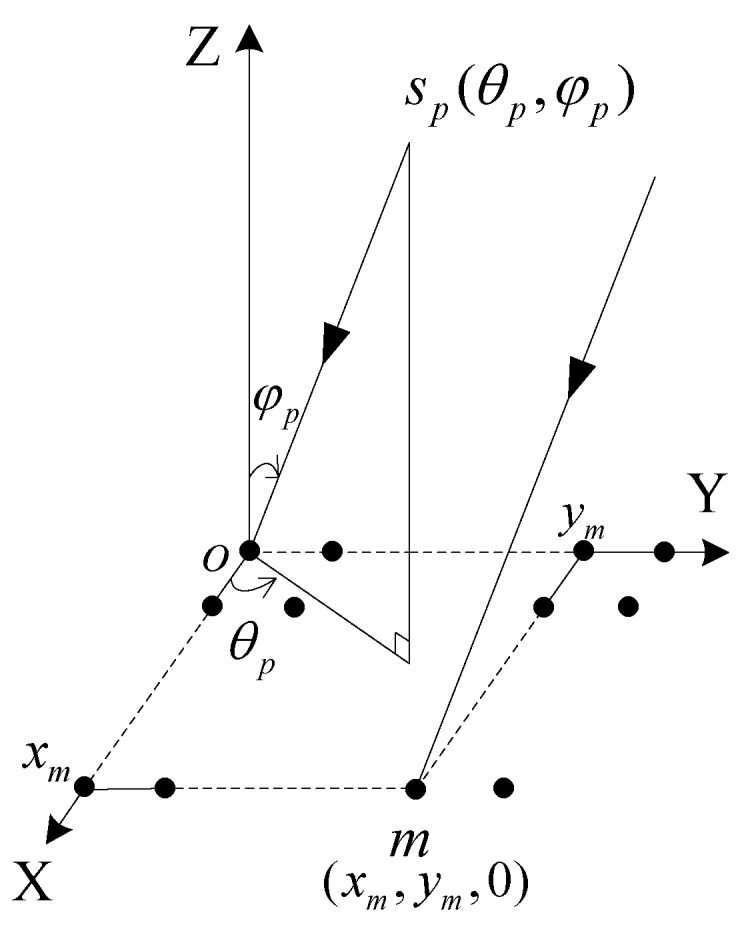
The coordinate system.

Consider a planar array composed of *M* (*m* = 1,2,…,*M*) sensors on which *P* (*p* = 1,2,…,*P*) narrow band non-coherent far field signals *s_p_*(*t*) are impinging with different DOAs. We denote the coordinates of the *m*-th sensor as (*x_m_*,*y_m_*,0), and the DOA related to the *p*-th signal is (*θ_p_*,*ϕ_p_*), where *θ_p_* and *ϕ_p_* are the azimuth and elevation, respectively. The uniform rectangular array (URA) coordinates system is shown in Figure 1. In the case of the *m*-th sensor receiving the *p*-th signal, there is a wave path difference between sensors. As illustrated by the coordinate system in Figure 1, if we choose the element at the origin of the coordinate system as the reference point, this path difference is calculated as:

(1) $$\Delta_{mp}=x_{m}\cos\theta_{p}\sin\varphi_{p}+y_{m}\sin\theta_{p}\sin\varphi_{p}$$

The complex factor corresponding to phase difference is calculated as:

(2) $$a_{mp}=\exp\;(-j2\pi \Delta_{mp}/\lambda )$$

where *λ* is the wavelength. At the *t*-th snapshot (*t* = 1,2,…,*T*), the signal arriving at the *m*-th sensor is:

(3) $$x_{m}(t)=\sum_{p=1}^{P}a_{mp}s_{p}(t)+n_{m}(t)$$

where *n_m_*(*t*) is the White Gaussian noise. The array output at the *t*-th snapshot is modeled as:

(4) $$x(t)={\left[x_{1}(t),x_{2}(t),\cdots ,x_{M}(t)\right]}^{T}=As(t\;)+n(t\;)$$

where *T* represents the transpose, **A** = [**a**_1_,**a**_2_,…,**a***_p_*] is the array direction matrix, **a***_p_* = [exp(−*j*2*π*Δ_1*p*_/*λ*),…,exp(−*j*2*π*Δ_Mp_/*λ*)]*^T^* is the steering vector, ***s***(*t*) = [*s*_1_(*t*),*s*_2_(*t*),…,*s_p_*(*t*)]*^T^* is the vector of source waveforms, and the noise vector **n**(*t*) = [*n*_1_(*t*),*n*_2_(*t*),…,*n_m_*(*t*)]*^T^* is assumed to be zero-mean and independent of the observed signal. The correlation matrix of the received data is defined as:

(5) $$R_{x}=E[x(t)x^{H}(t)]=AR_{s}A^{H}+\sigma^{2}I$$

where *σ*^2^ and **I** represent the noise power and unit matrix, respectively, and *H* represents the complex conjugate transpose. By the eigen-decomposition of **R***x*, we obtain the estimation of noise subspace ${\hat{U}}_{n}$. Utilizing the orthogonality between signal and noise subspace [25], the 2D MUSIC algorithm estimates the source DOA by a spectrum search as follows:

(6) $$P(\theta ,\varphi )=\frac{1}{\left|a^{H}(\theta ,\varphi ){\hat{U}}_{n}{\hat{U}}_{n}^{H}a(\theta ,\varphi )\right|}$$

where **a**(*θ,ϕ*) is the steering vector corresponding to the azimuth angle *θ* and elevation angle *ϕ*. When the noise subspace **U***_n_* is sufficiently accurate and (*θ,ϕ*) is equal to the actual DOA, the spatial spectrum function *P*(*θ,ϕ*) shows a peak. 2D MUSIC has to perform a 2D search with a certain stepsize over the angular field of view. A large stepsize may simplify the calculations but also lead to a worse RMSE. Furthermore, MUSIC possesses an extremely sharp spectrum peak, and a larger stepsize is more likely to miss the peak, especially in the case of a high signal-to-noise ratio (SNR). A small stepsize may improve RMSE, but may also increase computing overhead.

## 3. Spectrum Peak Diffusion Effect

From Equation (6), we can see that if signal or noise subspace is sufficiently accurate and the estimated $\left(\hat{\theta },\hat{\varphi }\right)$ equals to the actual (*θ,ϕ*), the spatial spectrum will show a peak. Usually, when $\left(\hat{\theta },\hat{\varphi }\right)$ deviates from the (*θ,ϕ*), there will still be a spatial spectrum peak although its peak becomes smaller with the increase of deviation from the actual DOA. We call this the spectrum peak diffusion effect. It is this observation that motivated us to change the traditional search strategy in 2D MUSIC, the diffusion spectrum peak provides us two pieces of information at least: one is that the actual DOA is near, and the other is the approximate direction of the sources.

**Figure 2:**
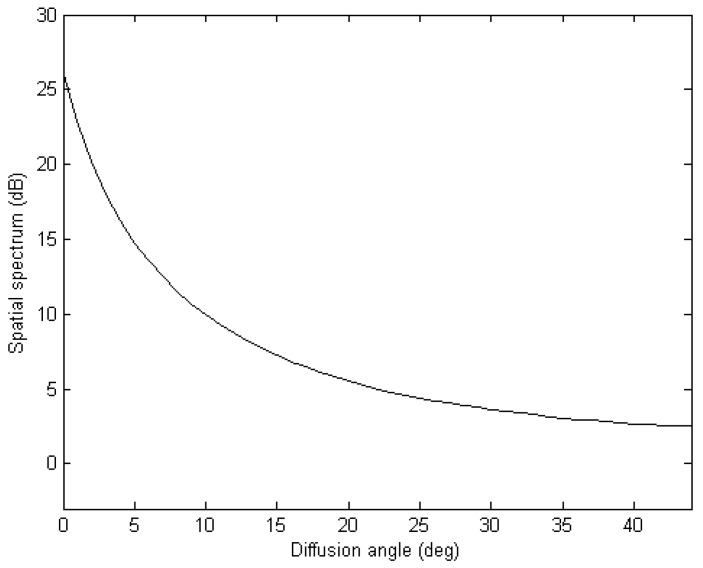
Spatial spectrum versus the diffusion angle.

Figure 2 shows the spatial spectrum versus the diffusion angle with 100 Monte Carlo trials. Here a uniform rectangular array with three rows and four columns omnidirectional sensors is used and a narrow band non-coherent far field signal comes from (45°,45°). The SNR, snapshot and stepsize are set to be 0 dB, 400 and 1°, respectively. The simulation in Figure 2 manifests that the spatial spectrum peak is high enough to be detected even the deviation is far from the actual (*θ,ϕ*).

## 4. Proposed Fast Algorithm

**Figure 3:**
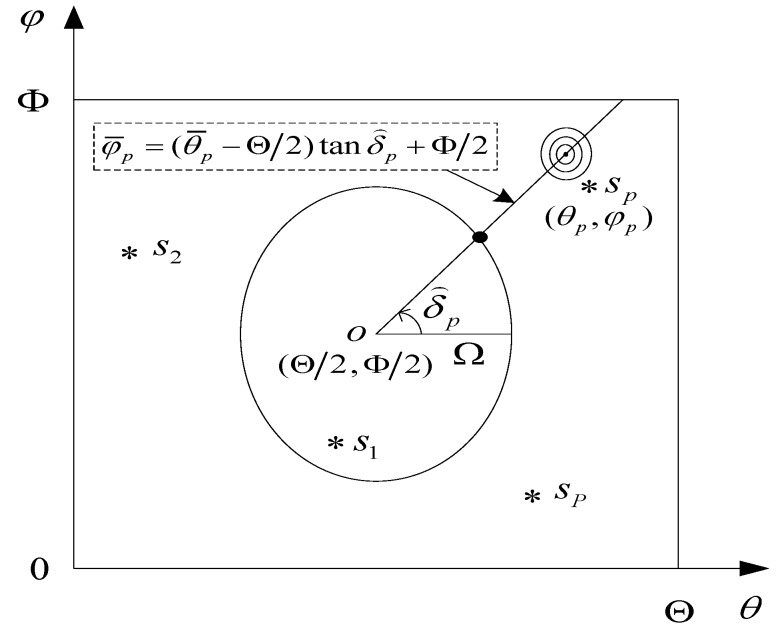
Proposed search scheme. *$s_{1}$ and *$s_{2}$ represent signals 1 and 2.

Suppose there are *P* narrow band non-coherent far field signals *s_p_*(*t*) (*p* = 1,2,…,*P*), the azimuth and elevation angle of the *p*-th source are set to be (*θ_p_,ϕ_p_*), where *θ_p_* ∈ (0°,Θ) and *ϕ_p_* ∈ (0°,Φ). Figure 3 shows the search scheme of the proposed AESS in the 2D DOA plane.

Our proposal is a three rounds search-based scheme. In the first search, we select the point *o*(Θ/2,Φ/2) as the center of a circle. Let Ω be the radius, which should not be too large or too small. An appropriate value should be selected for Ω in order to modestly sitting the circle on the area of 2D plane. It should be noted that the unit of Ω is not length but degree, so the parametric equation of this circle is:

(7) $$\left\{ \begin{array}{l} \theta =\Omega \cos\delta +\Theta /2\\ \varphi =\Omega \sin\delta +\Phi /2 \end{array}\right.,\;\delta \in (0{}^{\circ},360{}^{\circ}]$$

where *δ* is the central angle. The first search is implemented on this circle, since Ω is selected before the first round of search, it is actually a 1D search. The search variable is neither azimuth nor elevation, but the rotation angle *δ* which can be seen from Figure 3. The corresponding 1D spectral search is written as:

(8) $$P_{\mathrm{NO}-1}=\frac{1}{\left|a^{H}(\delta ){\hat{U}}_{n}{\hat{U}}_{n}^{H}a(\delta )\right|}$$

After searching on the circle with certain stepsize from *δ* = 0° to *δ* = 360°, and due to the existence of the spectrum peak diffusion effect mentioned above, we can get *P* spectrum peaks whose search variables are ${\hat{\delta }}_{p}$ (*p* = 1,2,…,*P*). Corresponding to each ${\hat{\delta }}_{p}$, we can get a straight line whose parametric equation is:

(9) $${\bar{\varphi }}_{p}=({\bar{\theta }}_{p}-\Theta /2)\tan{\overset{⌢}{\delta }}_{p}+\Phi /2,\;\left(p=1,2,\cdots ,P\right)$$

And the length of the *p*-th line (*p* = 1,2,…,*P*) is:

(10) $$L_{p}=\left\{ \begin{array}{l} \Phi /[2\sin({\overset{⌢}{\theta }}_{p})],\;\left|\tan{\overset{⌢}{\delta }}_{p}\right|>\Phi /\Theta \\ \Theta /[2\cos({\overset{⌢}{\theta }}_{p})],\;\left|\tan{\overset{⌢}{\delta }}_{p}\right|<\Phi /\Theta \end{array}\right.$$

Similarly, the unit of *L_p_* is degrees. The second round of searching is implemented along the *P* straight lines; it is also a 1D spectral search which is expressed as:

(11) $$P_{\mathrm{NO}-2}=\frac{1}{\left|a^{H}(l_{p}){\hat{U}}_{n}{\hat{U}}_{n}^{H}a(l_{p})\right|},\;l_{p}\in \left(0,L_{p}\right],\;\left(p=1,2,\cdots ,P\right)$$

Applying Equation (11), we can get one spectrum peak on each straight line. For each spectrum peak, the corresponding search variable in Equation (11) is marked as ${\tilde{l}}_{p}$. Now we can derive the azimuth and elevation $\left({\tilde{\theta }}_{p},{\tilde{\varphi }}_{p}\right)$ which corresponds to the spectrum peak on the *p*-th straight line, the parametric equation of ${\tilde{\theta }}_{p}$ and ${\tilde{\varphi }}_{p}$ is expressed as:

(12) $$\left\{ \begin{array}{l} {\tilde{\theta }}_{p}={\tilde{l}}_{p}\cos{\overset{⌢}{\delta }}_{p}+\Theta /2\\ {\tilde{\varphi }}_{p}={\tilde{l}}_{p}\sin{\overset{⌢}{\delta }}_{p}+\Phi /2 \end{array}\right.,\;\left(p=1,2,\cdots ,P\right)$$

Once the (${\tilde{\theta }}_{p},{\tilde{\varphi }}_{p})$ (*p* = 1,2,…,*P*) is adopted, we can perform the third round of searching which is a 2D search on a set of concentric circles, the centers of the circles are $\left({\tilde{\theta }}_{p},{\tilde{\varphi }}_{p}\right)$ (*p* = 1,2,…,*P*), respectively. This time the search variable are rotation angle *τ* ∈ (0°,360°] and the neighborhood radius is *ω*. The spectrum peak function is given as:

(13) $$P_{\mathrm{NO}-3}=\frac{1}{\left|a^{H}(\tau_{p},\omega_{p}){\hat{U}}_{n}{\hat{U}}_{n}^{H}a(\tau_{p},\omega_{p})\right|},\;\tau_{p}\in (0{}^{\circ},360{}^{\circ}],\;\omega_{p}\in \left(0,W_{p}\right]$$

Performing the above 2D search, we can obtain the search variable corresponding to the spectrum peaks which can be noted as $\left({\hat{\tau }}_{p},{\hat{\omega }}_{p}\right)$ (*p* = 1,2,…,*P*), from $\left({\hat{\tau }}_{p},{\hat{\omega }}_{p}\right)$ we can obtain the estimation of the actual 2D DOA as:

(14) $$\left\{ \begin{array}{l} {\hat{\theta }}_{p}={\hat{\omega }}_{p}\cos{\hat{\tau }}_{p}+{\tilde{\theta }}_{p}\\ {\hat{\varphi }}_{p}={\hat{\omega }}_{p}\sin{\hat{\tau }}_{p}+{\tilde{\varphi }}_{p} \end{array}\right.,\;\left(p=1,2,\cdots ,P\right)$$

## 5. Computational Complexity Analysis

We carefully compute the flops required by the classical 2D MUSIC and the proposed AESS methods. As mentioned above, *M*, *P* and *T* represent the total sensor number, source number and snapshot, respectively. Assume *K* represents the stepsize. Firstly, we present several basic operations and their computation consume in flops. Computing the multiplication of a *M* row and *P* column complex matrix by a *P* row *N* column complex matrix takes 6*MN*(2*P* − 1) flops. Computing the mathematical expectation of a *M* row *M* column matrix takes 6*M*^2^ + 1 flops. Computing the eigen-decomposition of a *M* row *M* column matrix takes 4*M*^5^ − 5*M*^4^ + *M*^3^ flops. Computing the absolute of a complex scalar takes 3 flops. Computing the reciprocal of a scalar takes 1 flops. 

For 2D MUSIC, assume the stepsize is *K*. Computing **R***_xx_* = *E*[**xx***^H^*] takes 8*M*^2^*T* − 2*M*^2^ + 1 flops. Computing **R***_xx_* = **UΣU***^H^* takes 4*M*^5^ − 5*M*^4^ + *M*^3^ flops. Computing $G_{N}={\hat{U}}_{n}{\hat{U}}_{n}^{H}$ takes 8*M*^3^ − 8*M*^2^*P* − 2*M*^2^ flops. Computing $P\left(\theta ,\varphi \right)=\frac{1}{\left|a^{H}\left(\theta ,\varphi \right)G_{N}a\left(\theta ,\varphi \right)\right|}$ takes (6*M*^2^ + 16*M* + 52)(Θ/*K*)(Φ/*K*) flops, where Θ and Φ are the maximum search scope of azimuth and elevation which are depicted in Figure 3. The total computation consumption in flops is:

(15) $$\begin{array}{cc} C_{2D\;MUSIC} & =(6M^{2}+16M+52)(\Theta \Phi /K^{2})\\ & +8M^{2}T+4M^{5}-5M^{4}+9M^{3}-8M^{2}P-4M^{2}+1 \end{array}$$

For the proposed AESS algorithm, the first three steps also involve computing **R***_xx_* = *E*[**xx***^H^*], **R***_xx_* = **UΣU***^H^* and $G_{N}={\hat{U}}_{n}{\hat{U}}_{n}^{H}$, so the corresponding computation consumptions are identical to 2D MUSIC. The following step of computing $P_{\mathrm{NO}-1}=\frac{1}{\left|a^{H}\left(\delta \right)G_{N}a\left(\delta \right)\right|}$ in Equation (8) takes (6*M*^2^ + 16*M* + 52)(360/*K*) flops. Computing $P_{\mathrm{NO}-2}=\frac{1}{\left|a^{H}\left(l_{p}\right)G_{N}a\left(l_{p}\right)\right|}$ in Equation (11) takes $\left(6M^{2}+16M+52\right)\sum_{p=1}^{P}\left(L_{p}/K\right)$ flops, where the meaning of *L_p_* can be seen from Equation (10).Computing $P_{\mathrm{NO}-3}=\frac{1}{\left|a^{H}\left(\tau_{p},\omega_{p}\right)G_{N}a\left(\tau_{p},\omega_{p}\right)\right|}$ in Equation (13) takes $\left(6M^{2}+16M+52\right)\sum_{p=1}^{P}\left(360/K\right)\left(W_{p}/K\right)$ flops, where the meaning of *W_p_* can be seen from Equation (13). The total computation consumption in flops is:

(16) $$\begin{array}{cc} C_{AESS}= & (6M^{2}+16M+52)\left[(360/K)+\sum_{p=1}^{P}\left(L_{p}/K\right)+\sum_{p=1}^{P}\left(360W_{p}/K^{2}\right)\right]\\ & +8M^{2}T+4M^{5}-5M^{4}+9M^{3}-8M^{2}P-4M^{2}+1 \end{array}$$

**Figure 4:**
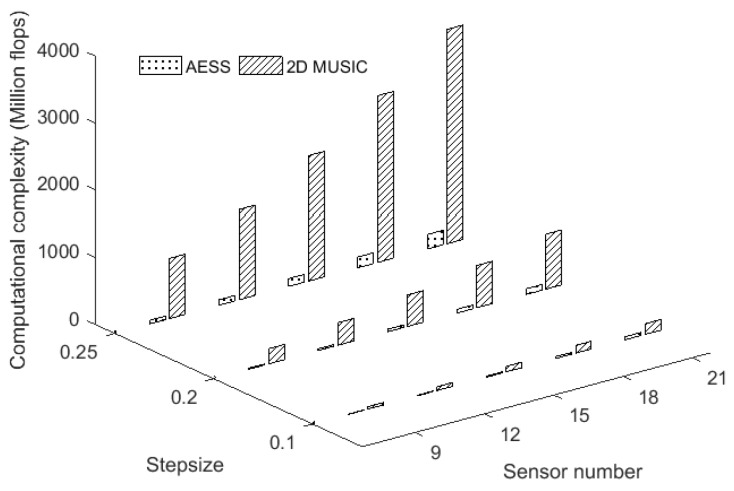
Computational complexity versus the sensor number and stepsize.

From Equations (15) and (16), we can see that the sensor number *M* and stepsize *K* are the two most important factors in determining the computational complexity. Snapshot *T* and the number of sources *P* are not the principal factors that increase the calculation. Figure 4 shows the computational complexity varies with the sensor number and the stepsize. We can see that the computational complexity of AESS and 2D MUSIC algorithms both increase with the decrease of stepsize. The increase of computational complexity of 2D MUSIC algorithm is much faster than that of the proposed AESS. AESS thus has a remarkable computational advantage over the conventional 2D MUSIC method, especially in the case of small stepsize. Filik [21] proposed a fast automatically paired 2D DOA estimation algorithm termed PIE, where they exploited the rotational matrix by employing real and virtual arrays, and deduced that the eigenvalues of the rotational matrix have the angle information at both magnitude and phase which allows the estimation of azimuth and elevation angles via closed-form expressions. Here we analyze the computational flops of the PIE algorithm with the symbols of [21]. Computing **y**_4_ = (**B**_12_ + **B**_13_)**y**_1_ takes 2*M*^2^ + 8*M*^2^*P* flops, computing $R_{y_{5}}=E\left[y_{5},y_{5}^{H}\right]$ takes 128*M*^5^ + 80*M*^4^ + 8*M*^3^ flops, computing $\hat{\Phi }={\left({\hat{S}}_{1}^{H}{\hat{S}}_{1}\right)}^{-1}{\hat{S}}_{1}^{H}{\hat{S}}_{4}$ takes 18*PM*^2^ − 6*PM* + 6*P*^3^+ (8/3)*P*^2^ flops, computing $\varphi_{i}=\arctan\left[\arg\left(\nu_{i}\right)/\arccos\left(\left|\nu_{i}\right|/2\right)\;\right]$ takes 9*P* flops, computing $\theta_{i}=\arcsin\sqrt{\left\{{\left[\lambda \;\arg\left(\nu_{i}\;\right)/2\pi d\right]}^{2}+[{\left(\lambda \;\arccos\left(\nu_{i}/2\right)/2\pi d\right]}^{2}\right\}}$ takes 27*P* flops. The total computation consumption in flops is:

(17) $$C_{PIE}=128M^{5}+80M^{4}+8M^{3}+\left(26P+2\right)M^{2}-6PM+6P^{3}+\frac{8}{3}P^{2}+36P$$

## 6. Simulation Results

In this section, simulations are presented to illustrate the performance of the proposed fast 2D MUSIC algorithm. A uniform rectangular array (URA) with omnidirectional sensors is used, along with three narrow band non-coherent far field signals coming from (20°,40°), (75°,50°) and (35°,60°).

### 6.1. RMSE and Estimation Success Rate versus Computational Complexity

The root mean square errors (RMSE) is defined as:

(18) $$\mathrm{RMSE}=\sqrt{\frac{1}{N}\sum_{n=1}^{N}\frac{1}{P}\sum_{p=1}^{P}\left\{{\left[{\hat{\theta }}_{p}(n)-\theta_{p}\right]}^{2}+{\left[{\hat{\varphi }}_{p}(n)-\varphi_{p}\right]}^{2}\right\}}\;(\mathrm{degree})$$

where *N* = 100 represents the independent trials, *P* = 3 represents the number of sources, ${\hat{\theta }}_{p}\left(n\right)$ is the estimation of *θ_p_* for the *n*-th Monte Carlo trial. The results have been averaged over 100 Monte Carlo runs.

**Figure 5:**
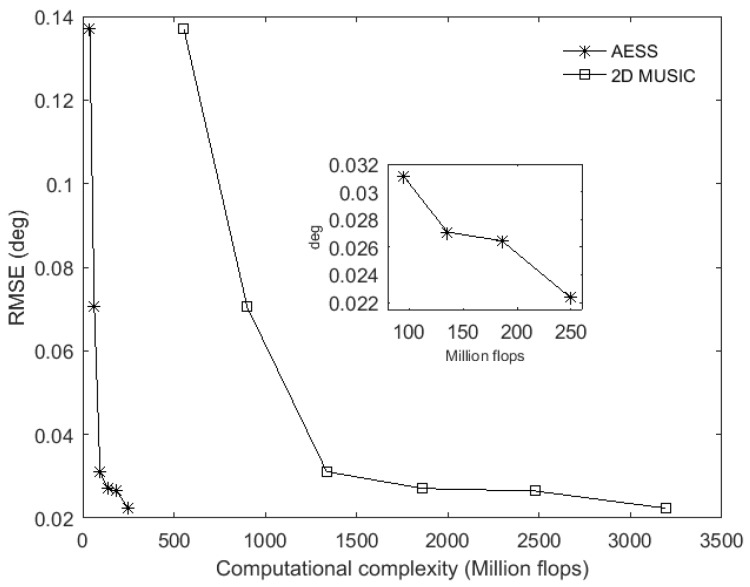
RMSE versus computational complexity.

It is obvious that the RMSE depends on the accuracy of the estimated subspace. Moreover, the estimated subspace is usually derived from the eigen-decomposition calculation. As this kind of calculation is critically affected by SNR and snapshots, the performance of RMSE is always closely related to the SNR and snapshots. In this paper, both the classic 2D MUSIC and the proposed AESS are search-based methods, they use the same subspace and their main difference is the search procedure, so SNR and snapshots have the same influence on RMSE. For this reason, our simulations here analyze the RMSE versus sensor number in equal stepsizes. We respectively use the rectangular array of three row and three column with a total of 3 × 3 = 9 omnidirectional sensors, and the following are 3 × 4 = 12, 3 × 5 = 15, 3 × 6 = 18, 3 × 7 = 21 and 3 × 8 = 24 omnidirectional sensors. From Equations (15) and (16), we know that the sensor number *M* and snapshot *K* take an important role in the computational complexity of the 2D MUSIC and proposed AESS, so substituting the value of sensor number into Equations (15) and (16) under a certain stepsize, we can get the corresponding computational complexity, hence we can derive the RMSE versus the computation complexity as shown in Figure 5. In Figure 5, SNR is set to be 0 dB, snapshot is set to be 400, and the stepsize is 0.1°. From Figure 5, we can see that if the stepsizes are equal, the classic 2D MUSIC and our proposed AESS possess similar RMSE, while the computational complexity of the proposed AESS will be greatly decreased. With equal computational complexity provided, AESS will derive a better RMSE than the 2D MUSIC algorithm. In this experiment, we also validate the DOA estimation success rate versus computational complexity, the SNR is fixed at 0 dB and snapshot at 400, stepsize is set to be 0.1°. In each independent run, we decide that the estimated $\left[{\hat{\theta }}_{p}\left(n\right),{\hat{\varphi }}_{p}\left(n\right)\right]$ are successfully resolved if:

(19) $$\left|{\hat{\theta }}_{p}(n)-\theta_{p}(n)\right|+\left|{\hat{\varphi }}_{p}(n)-\varphi_{p}(n)\right|\leq 0.5^{o}$$

**Figure 6:**
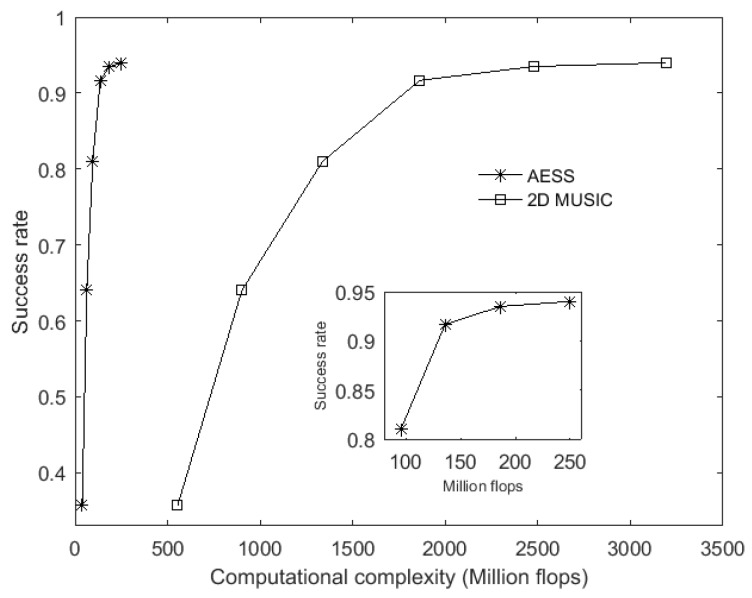
Success rate versus computational complexity.

Figure 6 demonstrates that the 2D MUSIC algorithm need many more calculation flops to obtain the same success rate compared to the proposed AESS. From another point of view, we can see AESS possesses much higher success rate in the same calculation flops provided. The numerical results show that with 39 to 250 million flops provided, AESS can cover the success rate from 36% to 94% while 2D MUSIC needs 554 to 3192 million flops to achieve the same success rate.

### 6.2. RMSE versus SNR and Snapshot

**Figure 7:**
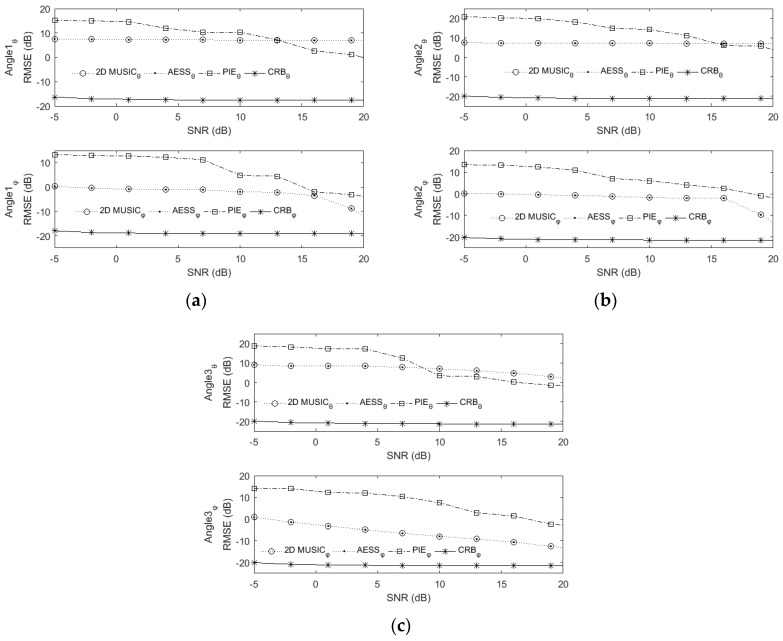
RMSE versus SNR. Snapshot is set to be 400, stepsize is set to be 0.1°. (**a**) The first source come from (20°,40°) ; (**b**) The second source come from (75°,50°) ; (**c**) The third source come from (35°,60°).

**Figure 8:**
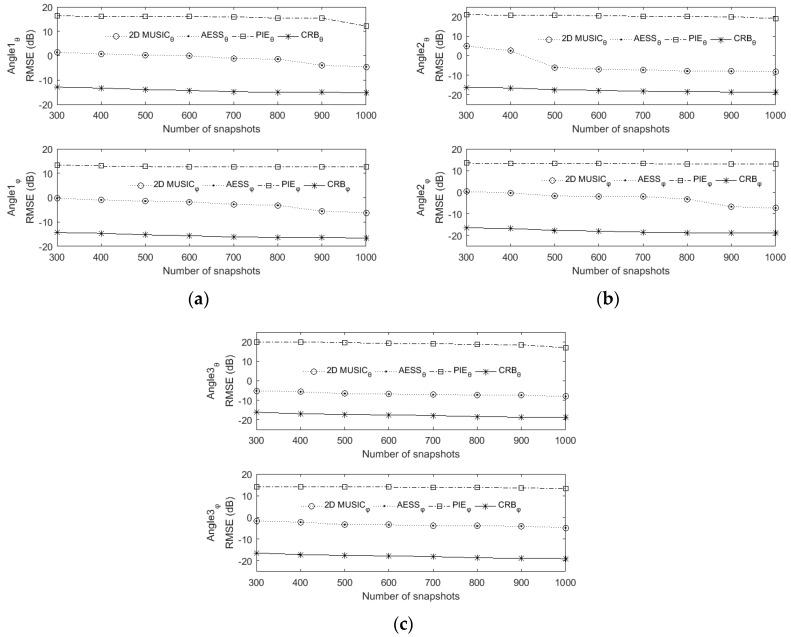
RMSE versus snapshot. SNR is set to be 0 dB, stepsize is set to be 0.1°. (**a**) The first source come from (20°,40°) ; (**b**) The second source come from (75°,50°) ; (**c**) The third source come from (35°,60°).

Figure 7 and Figure 8 describe the RMSE versus SNR and snapshot, respectively. Here a rectangular array of four rows and four columns with a total of sixteen omnidirectional sensors are used. In Figure 7 the snapshot is set to be 400, the SNR varies from −5 dB to 19 dB with 3 dB interval. 

In Figure 8 the SNR is set to be 0 dB, the snapshot varies from 300 to 1000 with 100 interval. The stepsize of 2D MUSIC and AESS in this experiment are all set to be 0.1°. The results have been averaged over 500 Monte Carlo runs. Since our proposed AESS and classic 2D MUSIC both are search-based methods, their RMSEs mainly depend on the stepsize, the two figures show that they possess almost the same RMSE versus SNR and snapshot. It should be noted that in the higher SNR scenario, the PIE algorithm derives a significant RMSE improvement.

### 6.3. Comparison Scatter Plots of Estimated 2D Angles in Different SNR

**Figure 9:**
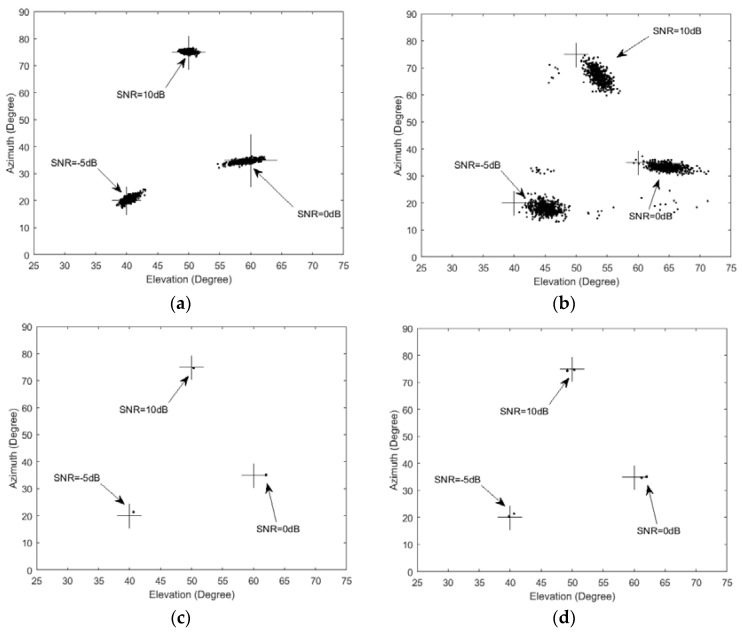
Scatter plots of estimated 2D angles when three sources with different SNR level are impinging on the URA. (**a**) Classic 2D MUSIC algorithm; (**b**) PIE algorithm; (**c**) The proposed AESS algorithm; (**d**) The proposed AESS algorithm with a slightly enlargement of the radius of the third round searching.

RMSE can coarsely reflect the estimated accuracy. For further analyzing the performances of the estimator, we show the scatter plots of the DOA in Figure 9. Here the SNR of three sources are set to be −5 dB, 0 dB and 10 dB respectively. A rectangular array of four row and four column with totally sixteen omnidirectional sensors are used. The stepsize of 2D MUSIC and AESS in this experiment are all set to be 0.1°. The snapshots of the three algorithms are set to be 400. The cross symbols in Figure 9 represent the actual position of sources.

Figure 9a–d demonstrate that a higher SNR makes each of the algorithms derive a smaller DOA estimation bias and smaller variance. On the other hand, lower SNR makes it hard to detect the DOAs. We can see that AESS has a higher uniformity than the 2D MUSIC and PIE algorithms. That is to say, its scatter plots concentrate on a smaller area than the other two algorithms. Unfortunately, the estimation is biased from the actual DOAs. The reason is that the last search of our AESS is implemented on several small concentric circles, when the actual source is situated outside the biggest concentric circle, AESS also take the highest spectrum peak inside the biggest concentric circle as the actual DOA. Obviously, this spectrum peak is the highest within the small area of concentric circles, but not the highest spectrum peak in the total angular field of view. This problem is inevitable for almost all local search methods. A slightly enlargement of the radius of the concentric circles can significantly decrease the estimation bias of AESS from the actual DOAs, which we can see from Figure 9d. The simulation conditions of Figure 9d are same as for Figure 9c except the search radius is modified from 5° to 7.5°.

### 6.4. CPU Time Comparison

In fairness to both algorithms, we compare the search time of the proposed AESS and the classic 2D MUSIC algorithm under same stepsize conditions. The results are obtained using a PC with an Intel(R) Core(TM) i5-3470 3.2 GHz CPU and 8 GB RAM by running the Matlab codes in the same environment. Table 1 shows that in the different stepsizes such as 0.1°, 0.25° and 0.5°, the search time of the proposed AESS is much smaller than that of the 2D MUSIC algorithm. It also can be seen that this advantage is more obvious with the decrease of stepsize. As PIE is a search-free method, it needs much less CPU time for the DOA estimation.

### 6.5. RMSE and CRB versus Source Separation δ

**Figure 10:**
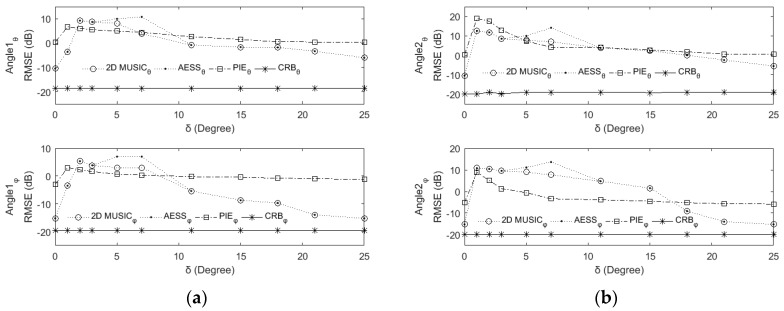
RMSE and CRB versus source separation. (**a**) RMSE of the first source; (**b**) RMSE of the second source.

To evaluate the resolution, we examine the capability of the proposed AESS and the related algorithms to estimate the DOAs of closely spaced signals. We fixed the first DOA source as (10°,40°), and the second source is situated at (*θ*_2_,*ϕ*_2_) = (10° + *δ*,40° − *δ*), where *δ* = 0°, 1°, 2°, 3°, 5°, 7°, 11°, 15°, 20°, 25°, respectively. From Figure 10 we can see that when *δ* = 0°, there is only one source and the RMSE is lower. When *δ* = 0°, 1°, 2°, 3°, 5°, 7°, the two source influence each other due to their close spacing, and the RMSE in this section are worse. When *δ* = 11°, 15°, 20°, 25°, the RMSE of all the algorithms are improved for the ease of distinguishing two far spaced signals.

### 6.6. Performance in the Presence of Mutual Coupling

**Figure 11:**
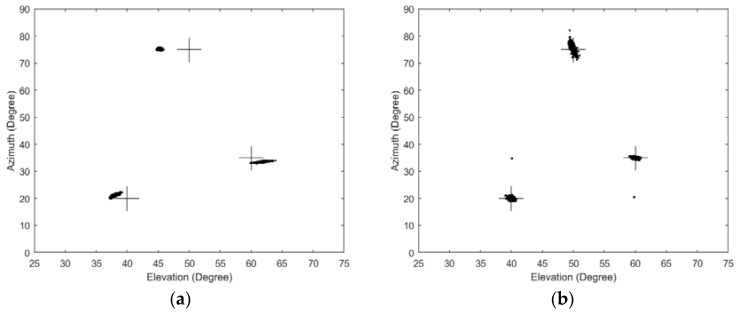

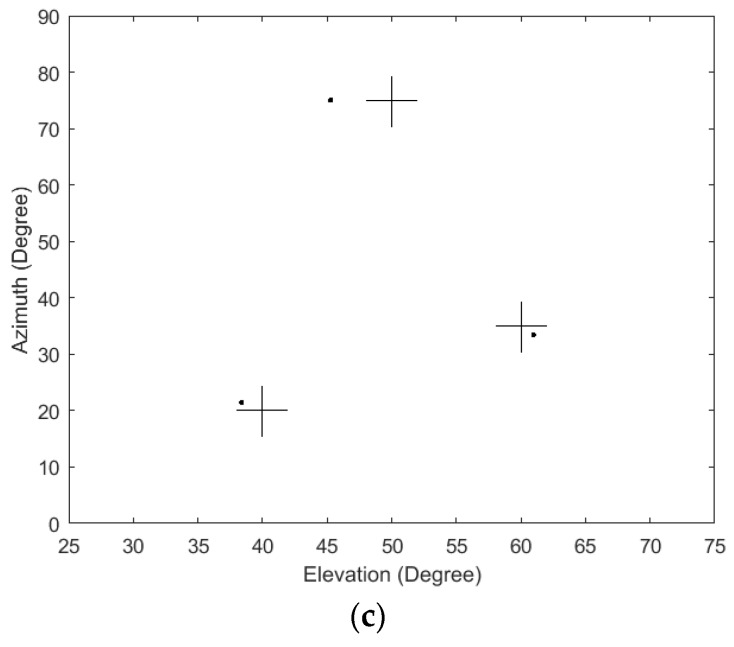
Scatter plots of estimated 2D angles in the presence of mutual coupling. (**a**) Classic 2D MUSIC algorithm; (**b**) PIE algorithm; (**c**) The proposed AESS algorithm.

As the DOA estimation performance is often affected by mutual coupling [18], we simulated the performance of our proposed and the related algorithms in the presence of mutual coupling. A rectangular array of four rows and four columns with a total of sixteen omnidirectional sensors is used. The stepsizes of 2D MUSIC and AESS in this experiment are all set to be 0.1°. The snapshots of the three algorithms are all set to be 400. The mutual coupling coefficient in our experiment is same as the literature [19], i.e., *c_x_* = *c_y_* = 0.3527 + 0.4854*i*, *c_xy_* = 0.0927 − 0.2853*i*. Scatter plots of the estimated azimuth and elevation are plotted in Figure 11, where the results have been averaged over 500 Monte Carlo runs. A challenge for further research is to incorporate the mutual coupling processing method to provide a more realistic scheme like the excellent algorithms [18,19,20] for applying sensor arrays.

## 7. Conclusions

We have proposed a new search-based algorithm, called AESS, for 2D DOA estimation. By exploiting the spectrum peak diffusion effect, our method is capable of searching the sources along their directions. Unlike the well-known 2D MISIC, which fixes one of the 2D angles and searches the other, the adjacent search points of AESS have different azimuth and elevation parameters to minimize the search path, which enables AESS to drastically reduce the computation and storage costs. Simulation results show that AESS achieves comparable RMSE and success rate but with a significant computational advantage over 2D MUSIC. Furthermore, the search process is much faster than that of 2D MUSIC in the scenarios of different stepsizes. We measured the RMSE versus SNR and snapshot, presented the CRB of each source for performance comparison with the theoretical optimum. To further evaluate the 2D DOA estimation performance, we presented the scatter plots of estimated 2D angles in different SNR, which show that AESS has smaller estimated variance although it is a biased estimation. Our simulation demonstrated that this bias can be decreased by slightly enlarging the search radius of the third round. To evaluate the resolution, we examined the capability of the proposed AESS and the related algorithms to estimate the DOAs of closely spaced signals. In the end, we tested AESS and the related algorithms in the presence of mutual coupling. A challenge for further research is to incorporate the mutual coupling processing method to provide a more realistic scheme such as the excellent algorithms in [18,19,20] for practical use.

## Figures and Tables

**Table 1:** Comparison of CPU time (s). Ss in the table means stepsize.

Configuration	Ss 0.1°	Ss 0.1°	Ss 0.25°	Ss 0.25°	Ss 0.5°	Ss 0.5°	
Row × Column	AESS	MUSIC	AESS	MUSIC	AESS	MUSIC	PIE
3 × 3	2.4032	41.9570	0.4205	6.6706	0.1228	1.7263	0.0043
3 × 4	2.4063	41.9736	0.4221	6.7262	0.1236	1.7394	0.0050
3 × 5	2.4176	43.4240	0.4228	6.8137	0.1238	1.7728	0.0052
3 × 6	2.4221	43.9129	0.4248	6.9126	0.1241	1.7955	0.0078
3 × 7	2.4399	44.8107	0.4398	7.0829	0.1351	1.8056	0.0089
3 × 8	2.5659	45.4299	0.4553	7.7317	0.1525	1.8943	0.0095
